# **18**β**-glycyrrhetinic acid inhibits rotavirus replication in culture**

**DOI:** 10.1186/1743-422X-9-96

**Published:** 2012-05-22

**Authors:** Michele E Hardy, Jay M Hendricks, Jeana M Paulson, Nicholas R Faunce

**Affiliations:** 1 Immunology and Infectious Diseases, Montana State University, PO Box 173610, Bozeman, MT 59718, USA

**Keywords:** Rotavirus, Licorice, 18beta-glycyrrhetinic acid, Antiviral

## Abstract

**Background:**

Glycyrrhizin (GA) and primary metabolite 18β-glycyrrhetinic acid (GRA) are pharmacologically active components of the medicinal licorice root, and both have been shown to have antiviral and immunomodulatory properties. Although these properties are well established, the mechanisms of action are not completely understood. In this study, GA and GRA were tested for the ability to inhibit rotavirus replication in cell culture, toward a long term goal of discovering natural compounds that may complement existing vaccines.

**Methods:**

Epithelial cells were treated with GA or GRA various times pre- or post-infection and virus yields were measured by immunofluorescent focus assay. Levels of viral proteins VP2, VP6, and NSP2 in GRA treated cells were measured by immunoblot to determine if there was an effect of GRA treatment on the accumulation of viral protein.

**Results:**

GRA treatment reduced rotavirus yields by 99% when added to infected cultures post-- virus adsorption, whereas virus yields in GA treated cultures were similar to mock treated controls. Time of addition experiments indicated that GRA-mediated replication inhibition likely occurs at a step or steps subsequent to virus entry. The amounts of VP2, VP6 and NSP2 were substantially reduced when GRA was added to cultures up to two hours post-entry.

**Conclusions:**

GRA, but not GA, has significant antiviral activity against rotavirus replication *in vitro*, and studies to determine whether GRA attenuates rotavirus replication *in vivo* are underway.

## Background

Rotaviruses are the most prevalent cause of acute viral gastroenteritis in children less than five years of age. Recent estimates indicate 2.7 million cases occur each year in the U.S. and 600,000 deaths occur annually worldwide (reviewed in [1]). Available data on two attenuated vaccines show they both are efficacious in reducing the incidence and severity of rotavirus gastroenteritis in the U.S. and other developed countries [2-5]. Reported vaccine efficacy is lower in developing nations where the majority of deaths from rotavirus infections occur, likely due to multiple factors including suboptimal immune responses associated with poor nutritional status or concurrent enteric infections [4,6,7]. Methods to enhance natural disease resistance find value in these regions if protective immunity could be improved by complementing existing vaccines with products that have antiviral or immune adjuvant activity.

Licorice is derived from the root of the perennial herb *Glycyrrhiza* spp*.,* and in addition to its use as a sweetening agent, has been one of the more extensively used medicinal plants [8]. Pharmacologically active components that have been most studied include triterpene saponins, with glycyrrhizin (GA) being present in the highest concentration [9,10]. 18β-glycyrrhetinic acid (GRA) is the aglycone product of GA hydrolysis mediated in the gut by bacterial glucoronidases [11,12]. Both GA and GRA have been studied in several systems to evaluate their immunomodulatory properties. GA has been used in Japan for >20 years to treat chronic viral hepatitis, and patients administered a continuous regimen of an intravenous formulation of GA (Stronger Neo--Minophagen C®, SNMC) demonstrate clinical improvement and reduced incidence of hepatocellular carcinoma [13-16]. GA also has been studied in an animal model of viral infection. Mice administered GA intraperitoneally survived a lethal dose of influenza virus [17]. Antibody to IFNγ abolished this protective effect, but the mechanisms of protection in this model system remain unclear. While mechanisms of action *in vivo* are not well understood, they likely are multi-factorial and could be due to direct effects on virus replication, or functions associated with modulation of inflammatory and cell protective responses.

The initial report describing antiviral activity of GA *in vitro* showed reduced replication of vesicular stomatitis virus (VSV), vaccinia virus (VV), herpes simplex 1 virus (HSV-1), and Newcastle Disease virus (NDV), but not poliovirus type 1 [18]. Subsequently, GA was shown to have antiviral activity against viruses in several families including flaviviruses, herpesviruses, influenza virus, SARS coronavirus, hepatitis C virus and others (reviewed in [19]). Suggested mechanisms of activity in these systems include direct effects on the adsorption, penetration and particle maturation steps of the replication cycle and in some cases, direct inactivation of virus particles. For example, anti-influenza virus activity of GA in cell culture was attributed to interference with viral endocytosis, likely due to its ability to modulate membrane fluidity [20,21], although alternative mechanisms recently have been proposed [22].

GRA has anti-tumorigenic, anti-ulcerative, anti-inflammatory, and anti-hepatotoxic activity *in vitro* and *in vivo*[19,23]. The utility of GRA as an antiviral compound is not as well described as GA, but there are data to suggest GRA has immunomodulatory and cell protective activity *in vitro* and *in vivo.* For example, *in vitro*, GRA induces NFĸB-mediated nitric oxide synthase expression in macrophages [24], and IL-8 expression in epithelial cells [25]. Others studies also suggest potential anti-inflammatory activity associated with reduced cytokine expression and attenuation of NFĸB activation [26,27]. *In vivo*, protective efficacy of GRA against protozoan and bacterial infections has been demonstrated. In a mouse model of visceral leishmaniasis [24,28], analysis of pro-inflammatory cytokine transcripts in isolated spleen cells from infected animals revealed that GRA induces IFNγ, TNFα, and IL-12, indicative of a T_H_1 and curative response. In a bacterial system, GRA attenuated lung pathology associated with *Staphyloccus aureus* pneumonia [29].

The relative lack of data regarding specific antiviral activity of GRA compared to GA led us to expand on previous data showing GRA treatment reduces rotavirus replication in cell culture [25], and include GA in our analyses, given the wealth of data available for this compound and its current availability for clinical use. We show that GRA treatment results in a significant reduction in rotavirus titers when added to cultures post-infection. The levels of viral structural proteins VP2 and VP6, and nonstructural protein NSP2 were reduced in GRA treated cells, consistent with anti-rotavirus activity of this compound.

## Results

### GRA and GA cytotoxicity in MA104 cells

**Figure 1:**
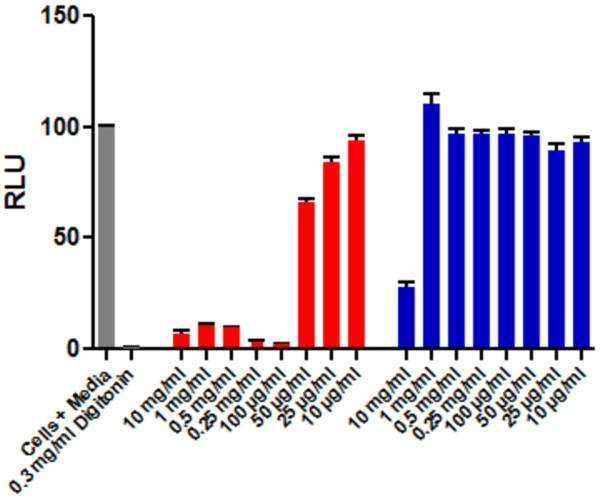
**GRA and GA cytotoxicity in MA104 cells MA104 cells were treated for six hours with the indicated concentrations of GA or GRA.** Viability was measured with the Promega CellTiterGlo Assay according to the manufacturer’s protocol, with digitonin as the control for 100% cytotoxicity. Red bars indicate GRA and blue bars GA. Data shown are representative of two experiments, with each concentration tested in triplicate in each experiment. Error bars indicate SEM.

We have shown that GRA is not toxic to MA104 cells up to the highest concentration tested of 10 μg/mL [25]. We performed an extended titration in this study and included GA, for which cytotoxicity in these cells had not previously been tested. MA104 cells were treated with serial dilutions of GA or GRA, and cytotoxicity was measured following six hours of incubation. The data shown in Figure 1 indicate that concentrations of 10 μg/mL and 25 μg/mL GRA showed only approximately 7% and 15% reductions in cell viability, respectively, at this time point. Extension of incubations past 24 hours resulted in significant cytotoxicity (data not shown). In contrast to GRA, GA was cytotoxic only at the highest concentration tested of 10 mg/mL. Each compound was used in the remainder of the experiments at a final concentration of 25 μg/mL.

### GRA reduces rotavirus infectivity while GA has no effect

**Figure 2:**
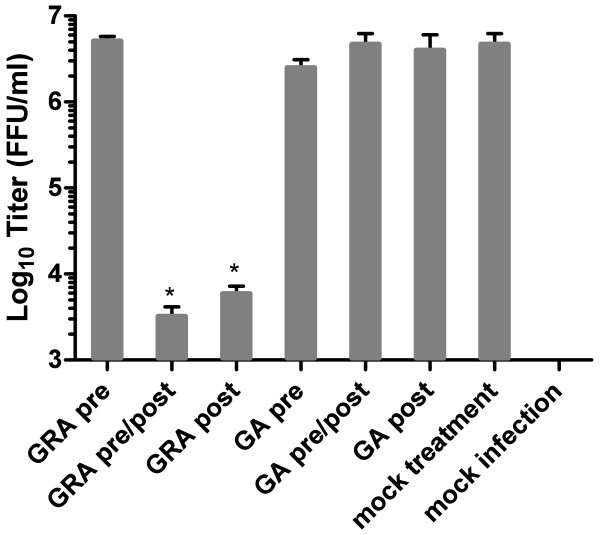
**GRA reduces rotavirus yields when added post--infection.** MA104 cells were infected with rotavirus strain RRV at a multiplicity of infection of 3 pfu/cell. 25 μg/mL GRA was added to the cultures either six hours pre-infection and then removed for the remaining time (pre) or was present continuously in the media throughout the course of infection (pre/post). For post-infection treatments, GRA was added to the cultures following the one hour virus adsorption period and maintained in the media throughout the infection. Total virus was harvested from cells and supernatant, and yields of infectious virus were determined by IFA. Data shown are representative of two experiments with triplicate samples in each experiment. Analysis for significance was performed using a two--‒tailed Student’s *t* test. Errors indicate SEM. *p = 0.006.

A previous time course of the effects of GRA on rotavirus replication showed an approximately 40% reduction in infectivity of bovine rotavirus strain NCDV when cells were treated as few as six hours prior to infection [25]. The prior experiments measured replication at a low multiplicity of infection (0.01 pfu/cell) over a long course of infection, and measured cell-associated virus. The current experiments were designed to measure one round of replication at a higher multiplicity of infection (three pfu/cell) so that we could begin to decipher mechanisms of inhibition that did not include cell-to-cell spread. To test inhibitory effects of GA and GRA on rotavirus infectivity, each compound was added to cells either six hours prior to RRV infection, or following the adsorption step. Cultures then were harvested six hours post-infection, and infectious virus was titered by IFA. In contrast to previous studies using a low multiplicity of infection, pre-treatment only did not significantly reduce virus titers under the conditions of these experiments (Figure 2). In contrast, GRA treatment resulted in a 99% reduction in infectivity when the compound was continuously present (pre/post) or when added post--infection only (post). This level of reduction in virus yields cannot be explained by simple cytotoxic effects on the cells at the degree defined by the toxicity assays. GA had no effect on virus yield whether the compound was added pre- or post-infection, as titers did not differ significantly from mock treated, infected controls. Therefore, GA was not investigated further.

### GRA does not reduce infectivity through a direct effect on the virus particle

**Figure 3:**
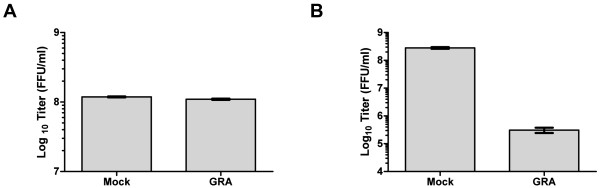
**GRA does not inactivate the rotavirus particle and inhibits replication postentry.** A) RRV stocks were incubated with 25 μg/mL GRA or DMSO for one hour at 37°C. Virus then was concentrated by ultracentrifugation for two hours at 35,000 rpm in an SW55 rotor (Beckman) and infectivity was measured by IFA. Data are from triplicate samples, p < 0.001. B) Trypsin‒activated RRV was inoculated onto cells at a multiplicity of infection of 3 pfu/cell and adsorbed for one hour at 4°C. Following adsorption, the medium was replaced and cultures were incubated for one hour at 37°C to allow virus entry. 25 μg/mL GRA or DMSO was added to the culture following the 37°C incubation, and virus titers following an additional 5 hours of infection were determined by IFA. Data were analyzed for significance with the two‒tailed Student’s *t* test. Error bars indicate SEM. p < 0.004.

GRA reduced rotavirus infectivity when added to the cultures following the adsorption step. To determine if there was a direct effect of GRA on the virus particle, RRV stock was mixed with 25 μg/mL GRA or DMSO and incubated for one hour at 37°C. Virus in suspension then was concentrated by ultracentrifugation and infectivity was measured by IFA. The data shown in Figure 3A illustrate that infectivity was not reduced when stock virus was treated with GRA prior to inoculation, suggesting that the ability of GRA to inhibit replication was not a result of a direct detrimental effect on the virus particle.

### GRA reduces infectivity at a post--entry step

GRA was inhibitory to RRV replication only when added to cultures post-virus adsorption. To determine whether the reduction in titer observed when cells were treated with GRA was a result of a block to virus entry, cells were inoculated with RRV at a multiplicity of infection of three pfu/cell, and virus was adsorbed for one hour at 4°C. Incubation at 4°C allows virus adsorption, but not entry into the cells [30,31]. After the adsorption period, cultures were incubated for one hour at 37°C to allow virus entry, and then GRA or DMSO was added and infections were allowed to proceed for five hours. The results showed that infectious virus titers were reduced by 99% when GRA was added following the 37°C incubation (Figure 3B), suggesting that GRA inhibits RRV replication at a step that is subsequent to virus entry into the cell.

### Viral protein levels are reduced in GRA treated cells

**Figure 4:**
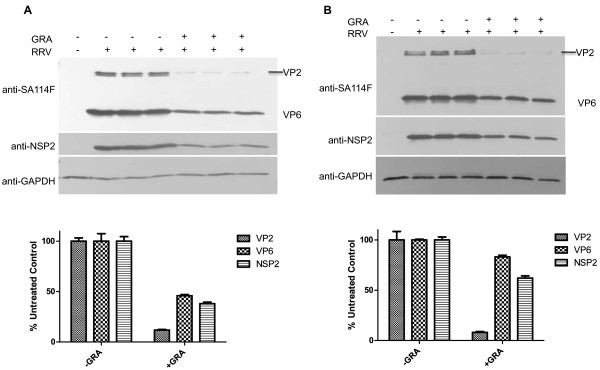
**GRA treatment reduces the levels of VP2, VP6 and NSP2 in infected cells.** Cells were inoculated with RRV at a multiplicity of infection of 3 pfu/cell. Virus was adsorbed for one hour at 4°C, and cultures then were shifted to 37°C for one hour (A) or two hours (B) prior to addition of GRA. Lysates were probed in immunoblots with anti-rotavirus strain SA11F antiserum to detect VP2 and VP6, anti‒NSP2, or anti‒GAPDH as a loading control. The lanes are triplicate samples. Bands were quantified by densitometry taking the averaged intensity of the bands in the individual samples normalized to the averaged intensity of the GAPDH loading control in each sample.

To begin to understand how GRA is inhibiting replication, the levels of viral protein in GRA treated cells were examined. As before, RRV was adsorbed to the monolayer for one hour at 4°C, adsorption medium was removed and replaced with fresh medium, and cultures were incubated for one hour at 37°C to allow virus entry. GRA then was added to the cultures and infections were allowed to proceed for five hours. Lysates were prepared and subjected to immunoblot. Figure 4A shows there was a substantial reduction in the amount of VP2 and VP6 in GRA treated cells. Evidence that virus replication was occurring and measurable at this time point was provided by detection of NSP2, the levels of which also were significantly reduced in treated cells compared to untreated cells. To further assess the reduction in the amount of VP2, VP6, and NSP2 in treated cells, the same experiment was performed, except that GRA was added two hours following the 37°C incubation. The results indicated that the degree of reduction in VP2 was similar to the previous experiment (Figure 4B). The amounts of VP6 and NSP2 still were diminished. However, they were higher than those observed when GRA was added one hour following the 37°C incubation. While one interpretation of these results suggests addition of GRA at later times post-adsorption may diminish its effects on levels of viral protein and consequently virus titers, the observation that the level of VP2 remained low suggests other mechanisms are at play. Together, the data show GRA has a profound effect on the accumulation of rotavirus proteins in infected cells, consistent with the observed reduction in yields of infectious virus.

## Discussion

The antiviral properties of two principal pharmacologically active constituents of licorice root, GA and GRA, have been extensively described, with by far the most data available for GA [8,23,32,33]. The mechanisms by which these compounds are antiviral, and the steps of the virus replication cycle at which they act vary depending on the virus under investigation. There are ample data to suggest antiviral activity of GA *in vivo* may result from induction of cytoprotective and immunomodulatory responses rather than effects on virus-specific functions per se, and such mechanisms of action could in part explain its broad-spectrum activity (reviewed in [32]. In contrast to GA, fewer reports of *in vivo* activity of GRA are available. However, when administered intravenously, GA is metabolized in the liver by lysosomal β-D-glucoronidase into 3-mono--glucoronide-glycyrrhetinic acid, and then finally by glucoronidases of intestinal bacteria into GRA, which then can be reabsorbed [11,12,34]. Therefore, it is possible that some of the *in vivo* effects of GA are due to one or more of its metabolites, including GRA.

We have shown that GRA reduces rotavirus yields during a single round of replication in MA104 cells whereas GA does not. Given numerous reports of antiviral activity of GA *in vitro*, it was somewhat surprising that GA did not effect rotavirus replication. However, inhibitory activity of GA may have a preference for enveloped viruses, consistent with potential mechanisms of action attributed to its effects on membrane fluidity.

GRA did not directly inactivate the rotavirus particle because yields of infectious virus were not reduced when virus stocks were incubated with the compound prior to inoculation onto cells. The reduction in yields of infectious virus and viral protein levels when GRA was added to cultures up to two hours post-adsorption suggests interference with rotavirus replication occurs at a step subsequent to virus entry. These data are consistent with previous studies illustrating a similar effect of GA on *in vitro* replication of NDV, VSV, HSV-1, and VV when the compound was added several hours post-infection [18]. Interestingly, the amount of VP2 present when GRA was added two-hours post-entry was similar to the levels observed when GRA was added one hour post-entry_,_ but the amounts of VP6 and NSP2 were considerably higher. This observation could be explained by effects on viral transcription, translation or degradation of viral proteins, but how such mechanisms would explain the selective reduction are difficult to predict. Studies to conclusively define steps at which GRA exerts its inhibitory effect are in progress.

There are multiple mechanisms by which GRA could reduce virus yields, including effects on specific steps of the replication cycle or on cellular pathways that play a role in establishment of an environment conducive to maximum replication efficiency. For example, we have shown that GRA induces NFĸB activation in MA104 cells [25], and more recently reported that some rotavirus strains down-regulate NFĸB activity through the functions of nonstructural protein NSP1 [35]. NFĸB activity is required for induction of a robust antiviral response, and sustained activation of NFĸB by GRA could override virus-encoded mechanisms that down-regulate its activity, resulting in reduced virus replication. Other studies also report GRA-mediated activation of NFĸB [24,28]. Of note, however, are reports showing GRA inhibits NFĸB activation, and this has been interpreted as GRA-mediated regulation of the inflammatory response [22,26,36]. In these and other studies, the effects of GRA on NFĸB were measured following induction of the pathway by virus infection or by pro-inflammatory mediators, suggesting that the effects of GRA on NFĸB are context-dependent. Deciphering the interactions between GRA and the NFĸB signaling pathway at the molecular level will contribute to understanding how its activity is modulated depending on the stimulus. One possible alternative mechanism of replication inhibition that is not mutually exclusive is provided by several reports indicating GRA modulates PI3/Akt activity [27,37,38]. Pharmacological inhibition of the PI3/Akt pathway substantially reduces rotavirus yields in culture [39]. The effects of GRA on these signaling pathways in the context of rotavirus infection and how GRA may modulate them currently are under investigation.

Together, the data reported here show GRA inhibits rotavirus replication at a step or steps subsequent to virus entry, and support antiviral activity of GRA reported in other systems. Rotavirus infects mature enterocytes at the villus tips in the proximal small intestine and thus *in vivo*, the tropism is polarized epithelial cells. Continuing studies will address whether there is a difference either in GRA toxicity or in the levels of reduction in virus replication in this cell type. We have administered GRA up to concentrations of 50 mg/kg to mice and have not observed clinical signs that would indicate toxicity (unpublished data), suggesting that inhibitory levels can be achieved *in vivo*. We currently are determining whether GRA-mediated replication inhibition *in vitro* translates to attenuation of virus replication in the mouse model of rotavirus infection.

## Methods

### Cells, virus, and compounds

MA104 monkey kidney epithelial cells (ATCC CRL2378) were maintained in Dulbecco’s Modified Eagle’s Medium (DMEM, Mediatech) supplemented with 5% fetal bovine serum (FBS, Atlanta Biologicals). Rhesus rotavirus strain RRV was propagated in MA104 cells and stock titers were determined by immunofluorescent focus assay (IFA, see below).

Glycyrrhizin (GA) and 18β-glycyrrhetinic acid (GRA) were purchased from Sigma-Aldrich. Stock solutions were prepared to a concentration of 100 mg/mL in DMSO and aliquots were stored at -80°C. Stock solutions were diluted to working concentrations in DMEM without FBS.

### Cytotoxicity assays

Cell viability under conditions of GA or GRA treatment was measured with the CellTiter-Glo Luminescent Cell Viability Assay (Promega) following the manufacturer’s protocol. Controls for 100% cell viability or 100% toxicity consisted of cells and media alone, or treatment with digitonin, respectively.

### Immunofluorescent focus assay (IFA)

Rotavirus infectivity following various treatments was determined by IFA as previously described [25]. Briefly, MA104 cells were cultured to confluence in 96-well microtiter plates. Wells were inoculated with 10-fold dilutions of RRV stock pre-treated with 10 μg/mL TPCK-trypsin (Worthington Biochemicals) for 30 minutes at 37°C. Sixteen hours post-infection, cells were fixed for 10 minutes with 2% paraformaldehyde, and then for 10 minutes with ice cold 100% methanol to permeabilize membranes. Structural protein VP6 was detected with mouse monoclonal antibody A6M [25] at a 1:200 dilution, followed by Alexa Fluor® 488-conjugated goat anti-mouse antibody (1:4000, Molecular Probes). VP6-positive foci indicative of infected cells were enumerated and titers expressed as fluorescent focus units (FFU) per mL.

### Infections, compound treatments, and culture harvest

Infections were performed as previously described [40]. MA104 cells were inoculated with trypsin-activated RRV at a multiplicity of infection of three pfu/cell, and virus was adsorbed to cells for one hour at 37°C. Following the adsorption step, media was removed and replaced with fresh media with or without compound according to the parameters of the experiment. Six hours post-infection, cells and supernatant were collected and subjected to three freeze-thaw cycles. Lysates were clarified by low speed centrifugation, and virus in the supernatant was titered by IFA.

GA, GRA, or DMSO vehicle control was added to cultures at the times indicated by each experiment. Pre-infection treatments were for six hours prior to addition of virus, and compound was not present in the media for the remainder of the infection. For post-infection treatments, compound was added following the virus adsorption or entry step and was present in the media for the duration of the infection.

### Immunoblots

Cell lysates for immunoblot were prepared as previously described [40]. Proteins were separated by SDS-polyacrylamide gel electrophoresis and immunoblots were probed with anti-SA114F rotavirus polyclonal antiserum that recognizes structural proteins VP2 and VP6, anti-GAPDH (Ambion), or anti-NSP2 (kindly provided by M Estes, Baylor College of Medicine) antibodies. Proteins that reacted with antibody were visualized by chemiluminescence using the Pierce ECL kit.

## Competing interests

The authors declare that they have no competing interests.

## Authors’ contributions

MEH conceived of the basic research premise, participated in experimental design and data interpretation and wrote the manuscript. JMH, JMP and NRF contributed to experimental design, performed experiments and assisted in manuscript preparation. All authors read and approved the final manuscript.
